# Addressing gaps in physician knowledge regarding transgender health and healthcare through medical education

**Published:** 2016-10-18

**Authors:** Deborah McPhail, Marina Rountree-James, Ian Whetter

**Affiliations:** 1 Community Health Sciences, Max Rady College of Medicine, University of Manitoba; 2 Max Rady College of Medicine, University of Manitoba; 3 Family Medicine, Max Rady College of Medicine, University of Manitoba

## Abstract

**Background:**

Transgender people (those people whose sex at birth does not “match” their felt gender identity) are a priority group for healthcare as they experience high rates of discrimination and related illnesses. Despite this, there is a trend of poor healthcare access for trans people due, in large part, to the denial of care on the part of physicians. A small body of literature is beginning to suggest that this denial of care may be due to a lack of physician knowledge as well as, in some cases, to transphobia. There is a dearth of research in Canada, however, exploring whether and/or how knowledge gaps create barriers to quality care, and whether medical education can attend to these gaps while and through addressing gender normativity.

**Methods:**

To fill these gaps in the literature, we undertook a qualitative study with 30 trans identified people and 11 physicians (*N*=41) in Winnipeg, Manitoba. Methods included semi-structured individual interviews and focus groups. Data were transcribed and analyzed with NVivo qualitative data software using iterative methods.

**Results:**

An overwhelming finding of this study was a lack of physician knowledge, as reported both by trans people and by physicians, that resulted in a denial of trans-specific care and also impacted general care. Transphobia was also identified as a barrier to quality care by both trans people and physicians. Physicians were open to learning more about trans health and healthcare.

**Conclusions:**

The findings suggest a pressing need for better medical education that exposes students to basic skills in trans health so that they can become competent in providing care to trans people. This learning must take place alongside anti-transphobia education. Based on these findings, we suggest key recommendations at the close of the paper for providing quality trans health curriculum in medical education.

## Introduction

Transgender people generally are defined as those who experience gender dysphoria, whereby the sex of one’s body at birth does not “match” one’s felt gender identity. As a result, some transgender people – though not all - may transition to the sex with which they identify through hormone treatments and/or a combination of surgeries called gender affirmation surgeries. Transgender people are a priority group for healthcare, with high rates of minority stress and related conditions such as suicide attempts, depression, and other mental illness.^1^–^3^ Research conducted thus far, however, shows a troubling trend of poor healthcare access for trans people. This poor access is related not only to an outright denial of care,^4^–^6^ as in the case of being turned away at the emergency room,^7^ but also to a denial of certain procedures specific to transitioning one’s sex, such as hormone therapy.^1^

The reasons for such denial of care seem to be two-fold. First, transphobia has been shown to impact care. In the United States, for example, Lambda Legal^8^ showed that mistreatment often occurred between healthcare professionals and trans people, including expressions of disgust on the part of the health professional, thus causing many trans people to avoid healthcare settings. In addition to transphobia, a lack of knowledge is suggested to be at the root of poor trans healthcare.^9^ Thus, while many physicians may wish to provide good care, studies also show that physicians often lack the knowledge to be in the position to provide it. Research suggests that doctors feel unprepared to treat health concerns related to transition care,^8^–^10^ even when such care falls within a physicians’ scope of practice, such as endocrinology or primary care.^8^–^12^

Overall, the literature suggests that trans people experience disparities both in health and healthcare, and that the disparities that they face in healthcare may be in part due to transphobia and to physicians’ lack of knowledge regarding trans health. Yet, though a growing area of inquiry, there is a dearth of qualitative research in Canada exploring how such a lack of knowledge is interpreted and experienced on the part of both trans people and, in particular, physicians (see Canadian context^8^ and US context^13^). Further, research is only beginning to explore how or whether this lack of knowledge can be addressed by medical education, and if addressing such knowledge gaps can alleviate the transphobia experienced by trans people at the hands of physicians. This paper begins to fill gaps in the literature by presenting the results of a qualitative study conducted in 2014–2015 that included indepth interviews with trans identified people about their healthcare experiences in Winnipeg, Manitoba, Canada, and focus groups with physicians. Unlike other studies,^8^,^13^ many of the physicians did not possess any sort of expertise in LGBTTQ (Lesbian, Gay, Bisexual, Trans, Two-Spirited, Queer) health. Thus, this paper is able to show the perspectives and knowledge gaps of a range of physicians. In the spirit of prioritizing the voices of trans people within discussions about trans care, we first outline the gaps in physician knowledge and transphobia in healthcare experiences as identified by trans participants. We then discuss how physicians approached these topics. We end by making recommendations for medical education and physician training, arguing that while addressing gaps in knowledge about trans health through medical education is important, only through addressing the internalized transphobia of learners will medical education about trans health be truly effective.

## Methods

### Study design, protocol, setting and sample size

The research was approved by the University of Manitoba Health Research Ethics Board.

We undertook a qualitative study in Winnipeg which included in-depth interviews with 30 trans individuals and three small focus groups with 11 physicians, for a total of 41 participants. Focus groups were physicians-only, while interviews were with trans individuals only. We undertook interviews with trans people, as opposed to focus groups, as we felt this format of a one-on-one conversation would allow for participants to delve as deeply as possible into their stories. We selected focus groups for physicians because of the fact that we wished to include participants with varying degrees of experience with trans healthcare. Focus groups can instigate discussions on topics that participants otherwise would perhaps shy away from given their perceived “non-expertise.”^30^ This proved to be the case in one focus group in particular, where physicians who were inexperienced with trans health were able respond to the topic by listening to and learning from participants with greater experience in the area. Less of such occurred in another group, however, where three residents appeared intimidated by the presence of their attending physician and spoke less freely than, perhaps, they would have otherwise.

Interviews with trans people were semi-structured, meaning that the interviewer asked a few guiding questions before giving participants room to “tell their own stories” and discuss issues of import to them. Physician focus groups were structured similarly and lasted up to two hours. Questions for trans people during the interviews were open-ended and centered on their experiences of healthcare as a trans-identified person, and whether or not and how these experiences could be improved. Questions for physicians focused on their experiences with trans people, barriers to providing good trans-focussed care, and how or whether medical education could address these barriers.

### Recruitment methods

Trans people were recruited by a variety of methods, including postering in clinics and other key gathering places, advertisements via Facebook and email listservs, and snowball sampling. Trans patient participants were given a $25 grocery store gift certificate in appreciation. Physicians were recruited through a number of means, including through snowball sampling and posters in a large, central downtown hospital and medical clinics. While we attempted to recruit a wide variety of physicians with various levels of knowledge about trans care, it is likely that our professional networks influenced participation response. While such “convenience sampling” may limit the outcome of the findings, in that the sample of physicians may not be as diverse as possible, such a limitation is generally acceptable in qualitative research.^29^ Rountree-James conducted all interviews and focus groups.

### Data analysis

The research team discussed emerging themes from interviews and focus groups as they occurred, and these discussions informed the ongoing research process as question probes were developed and changed slightly throughout the data gathering process. Interviews and focus groups were digitally recorded and transcribed by a professional transcriptionist. Transcripts were cleaned for identifying information during transcription and again during the coding process. Due to logistical issues, the data were coded after the data gathering process was complete. Therefore, while our interviews and focus groups were themselves iteratively adjusted based on the initial perceptions of the interviewer/focus group leader, our iterative analysis occurred solely with the interview transcripts. Specifically, the research team read transcripts repeatedly, and a code list was developed by McPhail and Rountree-James in response to themes that emerged during interviews and focus groups. Interviews and focus groups were coded using NVivo qualitative software by a research assistant in close consultation with the research team, and in particular with McPhail and Rountree- James who provided feedback with regard to the meaning and intent of each code. McPhail then undertook a thematic content analysis of the data,^15^ preparing code reports and writing memos and notes capturing the primary themes and sub-themes emerging from each code. The research team then validated and clarified themes in a community consultation session at the Rainbow Resource Centre in Winnipeg, attended by participants and other community members. Researchers also sought and received participant feedback via the distribution of a written report summarizing research findings.

### Participants

Trans participants ranged from ages 19–68. Sixteen of them identified as trans-women or female (transition from male to female), 12 as trans-men or male (transition from female to male), and two as gender non-conforming (identifying as neither male nor female). One psychiatrist, one surgeon, and nine family doctors participated in focus groups. Three of the 11 were residents, while the remaining were fully certified physicians.

## Results

### Trans patient experiences

Almost all trans participants reported encounters with physicians who displayed large gaps in knowledge regarding trans people and the healthcare they require. This often included transition-specific care, such as hormone prescription. As one participant related:

…lots of doctors that are practicing now they don’t have a clue what we’re talking about half the time. My doctor didn’t even know what level my T [testosterone] should be at.

Participants would sometimes describe the type of care denial noted by Beagan et al.,^8^ whereby their family physician felt the need to refer elsewhere for hormone prescription. Doctors would generally refer to Klinic Community Health Centre, the sole clinic in Winnipeg to specialize in transition care. One transman described:

…when I first went to my doctor, he was just not willing to help out. He preferred that I went to Klinic, right? I think his exact words were: “I have no issue with you being transgender or with the process. I’m just not familiar with it and I’d just prefer that you did everything with Klinic, because I’m just not experienced.”

While most participants did transition through Klinic, and were generally happy with that experience, they argued that relying on a specific clinic as opposed to having the ability to access generalized care created waiting lists at Klinic that, for people experiencing psychological trauma stemming from gender dysphoria, were sometimes unbearable. To solve this, one participant suggested better physician education regarding transition care:

There shouldn’t be just one [place] you can go to. …I think it’s just a matter of education so that everybody can be more competent and then trans people can just be streamlined into regular society, not marginalized to the degree that they are.

Gaps in physician knowledge were also evident in non-trans specific related care. For example, one participant, a trans-man, described seeing a gynecologist for a possible reproductive cancer. The gynecologist could not fathom treating a patient who identified as male:

There is just a lack of knowledge. [Gynecologists] are used to dealing with women who want to have babies or who are having some gynecological issue that they want to fix. And for me, I just wanted everything [all female reproductive organs] to be gone. I didn’t really care. …So, I, I had an appointment with [the gynecologist]. And she’s never met me before and was shocked to the point of – well, she left me in the room, by myself. …. She just, her face looked like I had just told her the most bizarre thing on the planet.

Another trans-man participant described an encounter with a physician who was shocked that he had been pregnant, even after the participant had explained to him that he had transitioned:

…[the doctor] just couldn’t understand it. He had some kind of block. …I told him that I had been diagnosed with postpartum psychosis. And he was like “How could you possibly have been pregnant?” Well, it’s not that hard to understand.

Echoing findings in the US,^9^ this lack of knowledge and familiarity on the part of doctors often created the need for people to educate doctors not only about what trans people were, but also about what kinds of healthcare needs they might have; this could lead to deep resentment. As one participant stated:

I feel always with going to healthcare practitioners, I have to educate people all the time. And I feel really resentful of that.

Other times, participants appreciated that while doctors were not well educated about trans health, they were willing to ask questions from their patients in order to learn:

[My doctor] did not know very much about transgender health at all. So she’ll ask me questions and I’ll answer as best I can. Which I think is great. Because she’s not afraid to ask me.

Importantly, while many participants expressed appreciation for physicians who were willing to ask respectful questions, they cautioned against asking unnecessary and invasive questions unrelated to the medical condition at hand. One participant was asked, while in emergency on an issue completely unrelated to his genitals, whether or not he had “had the surgery” yet. This participant noted:

You don’t need to know that if you’re just giving me a few pills. …[It was] this curiosity thing and I was very tired and hungry and vulnerable in that situation. It felt very invasive.

For the most part, then, even though it would cause resentment at times, doctors’ lack of knowledge about trans health was not always regarded as discriminatory. The genuine lack of knowledge about trans-people and trans health on the part of physicians, was not always regarded as intentionally hurtful. As one participant said: “[there are] a lot of really well meaning and well intentioned people, just not well versed…[they’re] not intentionally transphobic.” There were other times, however, that, participants certainly described instances of discrimination in encounters with physicians and allied healthcare professionals.

The excessive and unnecessary questioning about genitals described above was regarded as transphobic in that it felt “othering.” Participants also regarded the consistent use of incorrect gender pronouns, despite correction, as disrespectful and transphobic. Additionally, participants described general instances in which a healthcare provider communicated extreme lack of comfort with trans people by, for instance, not looking them in the eye or ending the clinical encounter as quickly as possible. For example, one participant, who identified as trans and non-binary (and used the pronoun “they”), described their experience of emergency care. Important to this story is to note that this participant wore a binder, which is an elastic cloth that flattens one’s chest for a more masculine appearance:

I went to…emergency. And finally saw a doctor and I’m sitting on the table and he’s like “Okay, so what’s wrong?” And I was like “I was queer bashed last week and I think I have bruised ribs. I wear a binder. And I feel like it’s making it worse and I don’t really know. What my other options are or what other treatments there are? Because I’m not going to stop wearing it.” And he paused and was like “Okay, so then you could just stop wearing it then, right?” And I was like “No, no, no. I just said when I go out in public, I can’t, I don’t, I don’t feel comfortable not wearing it.” And then he just kind of stared at me for a while. And it was a weird kind of stare. And there was this weird distance. …I walked out of there with a prescription for 60 T3s. Who gives 60 T3s? Who gives 60 T3s to someone to begin with? The answer is someone that’s really freaked out with you and just wants you to leave.

Thus, mirroring previous studies demonstrating systemic transphobia in ER settings,^7^,^27^ the trauma of being queer-bashed for this participant was exacerbated by the identity-based violence they experienced in the hospital at the hands of a doctor who felt profoundly uncomfortable with their trans/non-binary embodiment.

### Physician perspectives

In line with the literature, physicians in the current study expressed little knowledge about trans care. Often, participants felt a real concern that their knowledge deficit could harm patients. As one physician explained:

For me there’s this fear that run of the mill problems aren’t run of the mill. What if there’s something related to something I’m not aware of, in terms of their hormonal status, in terms of the medications they’re taking? ...I have a lot of anxiety seeing these people, not because of who they are, but because I feel I’m not well educated, I’m not well prepared about what the potential concerns are.

In general, physicians described gaps in their education regarding hormone prescription and monitoring. Physicians also demonstrated a lack of knowledge regarding gender dysphoria and how to diagnose it so that a patient can begin the transition process. Additionally, some physicians were struggling with language and interpersonal communication with trans people. One physician noted:

…the hardest thing for me is the [gender pronoun] designations and “he” and “she.” And then there are a group of people who don’t want to be identified as either.

Physicians varied from having zero exposure to transgender education during their training to having a day of LGBTTQ education every year plus student-driven interest groups that allowed students to learn more and have greater exposure to LGBTTQ medicine and patient care issues. Physicians had some differing ideas about curriculum content in medical education, suggesting formats such as small group learning, self-learning, standardized patient role play situations, and real in-clinic exposure. One physician suggested that trans people could be integrated into case-based learning so as to normalize and include education on treating trans patients: “If it’s incorporated into studying [other topics] then it’s just part of what you learn.” While ideas about *how* to learn trans health varied, many agreed that learning about the provision of quality care early on in training is positive. One physician argued:

I think there is value to having one short trans health session in med 1 or med 2, mainly to put it on people’s radars. It’s not that anybody’s going to be an expert in trans health after an hour, but the idea that there are people who need this service is a valuable seed to plant... It was not on my radar screen. It wasn’t that I was opposed to it. It wasn’t that I wasn’t open to it. It didn’t occur to me until I was in practice.

Thus, most of the physicians agreed that more education on trans health was necessary. A small number also discussed transphobia as a barrier to care. For example, when asked what they thought were major health issues for trans people, a participant in Focus Group 1 replied: “accessing healthcare without stigma.” Similarly, a participant in Focus Group 3 who had a high degree of experience in trans healthcare, related a story about a transphobic professional in her clinic, who was discriminatory despite having had training and education. Within the context of this story, the participant noted:

It’s just, it’s so rooted, culturally, gender is such a core part of people’s identity. A newborn baby, the first question everybody asks is ‘Is it a boy or a girl?’ sometimes before the baby’s born, right? So, that’s such a core part of, of people’s identity, that it’s hard to wrap your head around that not necessarily having to be a fixed thing. You have to think about your language and you have to think about that sort of thing. And that’s, it’s tricky. And it’s not that it’s impossible. It’s not that it can’t be done. It’s just, you kind of just have to think. The whole evolution of this [discussion]…you know, [it has to occur] society wise. Because we’re all, I know I’m a product of society. …The first thing I ask is, ‘Is it a boy or a girl?’ too, right?

Despite most participants’ focus on education, then, the above quotation points to the questionable efficacy of educating students about competent trans healthcare within the context of a society fixated on gender binarism, in which all learners are, of course, embroiled.

On the other hand, while some participants recognized the importance of addressing transphobia in healthcare, whether through education or through a more systemic, macro approach, a handful of participants questioned the existence of transphobia in healthcare at all. For example, one physician stated:

There’s a perceived fear of there being a barrier. There’s a perceived social stigma that [trans people] have. And I don’t know if that ah, has any reality in Winnipeg, in that doctors would be prejudiced against them in any way, shape or form. I don’t know if that fear is founded in reality or just in their experiences or perceptions.

Along similar lines of questioning the legitimacy of trans people’s experiences of discrimination in healthcare, another participant argued that trans people should take responsibility for instances of mis-naming: “I personally feel like they have some responsibility to, if this is, if they’re going to be upset to be called the wrong name, that they should change it officially. Cause this is part of the problem.”

## Discussion

One of the key gaps in care identified in our study by both trans patients and physicians was a lack of knowledge on the part of physicians regarding trans health and healthcare issues. Overall, many doctors with whom trans people interacted were well-intentioned, but simply did not know about trans people and/or trans health, thus causing them to deny care. When doctors did not feel competent with transition care, they would often refer to specialists in trans care, causing significant wait lists. Given high rates of suicide that have been shown to occur whilst awaiting transition care,^25^ such a long waitlist is a significant and pressing problem that needs to be addressed. Further, the turning away of patients as described by trans participants because of a lack of knowledge about relatively basic medical procedures and care provision is, from our perspective, unacceptable. One logical solution to this lack of knowledge is better preparation of future physicians in medical school. In the allied health professions such as pharmacy,^17^ occupational therapy,^10^ and nursing,^18^,^19^ literature has reported positive change after the introduction of trans-specific curriculum to undergraduate education that centered around the distinctions among sexual orientation, sexual behaviours, and gender identity, as well as how to ask about and discuss these topics in a respectful way.^20^ The lack of education during medical school on how to treat transgender patients has been well documented, however.^21^–^24^ We suggest that these knowledge gaps can be addressed through better medical education that exposes students to basic skills in trans health so that they can become competent in providing care to trans people.

At the same time, as one physician in the study noted, education alone that simply fills gaps in knowledge without addressing the systemically socialized transphobia of healthcare professionals - instances of which were described by many trans participants and demonstrated by the physicians in the study who denied and de-legitimized transphobic experiences - will not likely be effective. Thus, as Beagan et al. have argued previously in this journal,^26^ while medical education about trans health is integral to interrupting transphobia in healthcare settings, such education cannot focus on “facts” about hormone values or surgical after-care alone. Medical education must also examine and address the “attitudes and perceptions” of students, so that they might “understand that social messages are internalized inadvertently… [in order to] counter them.”^26^^(p.e20)^ To do so, we would argue with Baker and Beagan^28^ that students must experience cultural humility and discomfort^29^ through curricular activities, in which they have a safe space to reflect upon their power, privilege, and their deeply held beliefs about gender normativity.

Thus, based on our research and given the literature, we would recommend that curricular programs in medical schools be implemented that emphasized:

1. A very basic overview on what “transgender” means and the health issues of trans people.
2. Training in language and proper pronoun use for interactions with patients.
3. Basic training in the area of hormone prescription and monitoring hormones over time.
4. Basic training in the diagnosing of gender dysphoria in the clinical setting.
5. Discussions regarding proper referral for surgical procedures.
6. A varied pedagogical approach that includes lectures from medical experts in trans health, community panels, case studies, clinical exposure, role play, and standardized patient interaction.
7. Content that is delivered in specific sessions concentrating exclusively on trans health, and that is also interwoven throughout *all* of the medical curriculum in the form of case studies, for example.
8. Reflective exercises, small group activities, and lectures that allow for students to reflect upon their preconceived notions about gender and gender norms and the privileges and power that students themselves exercise as a result of systemic transphobia.
9. The systemic nature of transphobia in society, and how this founds and produces transphobia in the healthcare setting.

### Conclusions

This study is limited by its relatively small sample size and, as such, is not generalizable. Further, it incorporates a self-selected sample. The study does provide, however, further evidence to suggest transphobia and major gaps in physician knowledge regarding trans health and healthcare, and demonstrates how patients experience these issues that can and should be addressed through medical education. Further, our research suggests that physicians are not necessarily averse to training of this nature, but in fact would welcome more knowledge about trans people and how to meet their healthcare needs with compassion and respect.
